# Swedish study participants undergoing research bronchoscopy – a tolerable or unpleasant experience?

**DOI:** 10.3389/fmed.2025.1648729

**Published:** 2025-09-12

**Authors:** Pernilla Sönnerfors, Petra Kristina Jacobson, Anders Andersson, Leif Hilding Bjermer, Anders Blomberg, Heléne Blomqvist, Christer Janson, Jonas S. Erjefält, Hans Lennart Persson, Ellen Tufvesson, Åsa M. Wheelock, Carl Magnus Sköld, Annelie Behndig

**Affiliations:** ^1^Department of Medicine Solna, Division of Immunology and Respiratory Medicine, Center for Molecular Medicine, Karolinska Institutet, Stockholm, Sweden; ^2^Women’s Health and Allied Health Professionals Theme, Karolinska University Hospital, Stockholm, Sweden; ^3^Department of Respiratory Medicine in Linköping, Linköping University, Linköping, Sweden; ^4^Department of Health, Medicine and Caring Sciences, Linköping University, Linköping, Sweden; ^5^COPD Center, Department of Respiratory Medicine and Allergology, Sahlgrenska University Hospital, Gothenburg, Sweden; ^6^COPD Center, Department of Internal Medicine and Clinical Nutrition, Institute of Medicine, Sahlgrenska Academy, University of Gothenburg, Gothenburg, Sweden; ^7^Department of Clinical Sciences, Respiratory Medicine, Allergy and Palliative Medicine, Lund University, Lund, Sweden; ^8^Department of Public Health and Clinical Medicine, Umeå University, Umeå, Sweden; ^9^Department of Respiratory Medicine and Allergy, Karolinska University Hospital, Stockholm, Sweden; ^10^Department of Medical Sciences, Respiratory, Allergy and Sleep Research, Uppsala University, Uppsala, Sweden; ^11^Unit of Airway Inflammation, Department of Experimental Medicine Sciences, Lund University, Lund, Sweden

**Keywords:** research bronchoscopy, participants’ experiences, airway sampling, research setting, patient information

## Abstract

**Introduction:**

Flexible bronchoscopy is regarded as a safe examination and is commonly used in the diagnostic work-up for lung diseases, but is also important in pulmonary research. We aimed to investigate participants’ experiences when undergoing bronchoscopy in a research setting.

**Methods:**

Participants were recruited from the Swedish CArdioPulmonary bioImage Study (SCAPIS). A subset from this cohort (*n* = 45, mean age 60.5 years, 20 with normal lung function and 25 with chronic obstructive pulmonary disease, COPD) was selected for bronchoscopy. The procedure was explained both orally and in writing during a pre-procedure visit. The information included premedication, monitoring, local anesthesia, airway sampling [bronchoalveolar lavage (BAL), bronchial wash, and mucosal biopsies], and urine and blood samples. Questionnaires pre- and/or post-procedure were used to assess experiences and health impacts.

**Results:**

In general, participants found the bronchoscopy procedure acceptable and only a few (18%) found it unpleasant. A majority (80%) reported their experience to be much better or as expected. Almost all participants (93%) were very satisfied with the information provided. Topical anesthesia was seen as more unpleasant (20%) than airway sampling (11%). Notably, more women and participants with normal lung function reported BAL as unpleasant. After the procedure, chills, fever, and hemoptysis were reported, but no serious adverse events occurred. Increased cough and phlegm were noted.

**Conclusion:**

The present study, conducted by experienced bronchoscopists and healthcare teams, demonstrates that a bronchoscopy in a research setting in well-informed participants with normal lung function or COPD was well-tolerated.

## 1 Introduction

Flexible bronchoscopy is regarded as a safe examination method and is routinely used in the diagnostic work-up of lung diseases, but it is also important in pulmonary research. The side effects of bronchoscopy are primarily related to the procedure itself, such as bleeding, cough, and oxygen desaturation, but fever can also occur (1–4). The willingness to participate in research bronchoscopy is often high in resource-limited settings, due to the possibility of getting a free health check and showing gratitude to society (5). Younger age (<40 years), higher education level, a prior history of donating blood and having a chronic disease have been reported to increase the willingness to participate. The possibility of finding new treatments in the future has also been shown to increase motivation (6). However, it has also been shown that financial reward and altruism were highly motivating factors for healthy participants to participate in research studies (7). In a high-income setting, participants with chronic obstructive pulmonary disease (COPD) and asthma have also expressed a wish to help others and a hope to contribute to science. Also, personal benefits and a subjective feeling of being obligated to participate have been reported as factors (8). Well-trained recruiters, who can provide the participants with correct and trustworthy information, are essential, as trust in the researchers is an important factor in increasing the willingness to participate in medical research. Perceived risk of harm and fear of discomfort are major reasons for refusal to participate in health research (6, 8). Predictors of patients willing to participate in a second bronchoscopy are lower age, higher education, use of higher doses of anxiolytics and an inpatient setting (9).

Little is known about participants’ experiences of the procedures to undergo a bronchoscopy in a research setting, including their well-being on the following day. Knowledge of participants’ experiences of taking part in a research bronchoscopy might help optimize the recruitment process and the participant rate. Furthermore, to guide researchers in how to prepare participants and what to include in comprehensive information regarding the procedure might improve the overall experience. This study aimed to investigate the experiences of middle-aged participants with normal lung function and with COPD undergoing a bronchoscopy in a research setting.

## 2 Materials and methods

### 2.1 Study subjects

**Figure 1:**
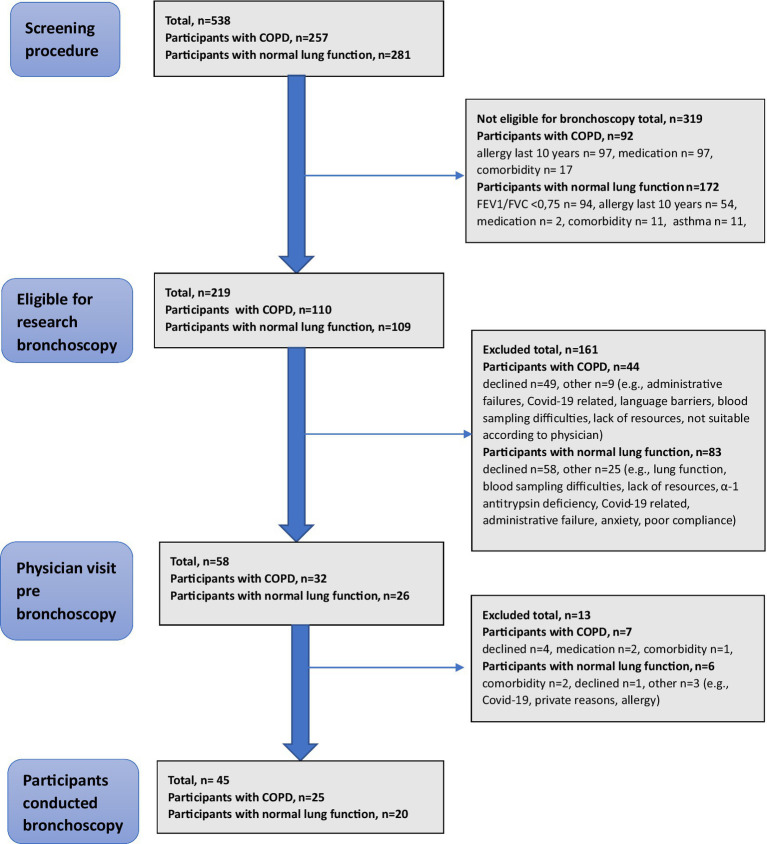
Flowchart of participants included in the BRONCHO-SCAPIS study undergoing research bronchoscopy.

The study was based on the Swedish CArdioPulmonary bioImage Study (SCAPIS), a multi-center study investigating a population-based sample of the general Swedish population (*n* = 30,154) (10). Participants aged 50–75 years were screened and recruited based on smoking history and lung function to join the BRONCHO-SCAPIS study (*n* = 690)^1^ conducted at six university hospitals in Sweden from 2017 to 2023, with the primary aim to study COPD in never-smokers (11). In the present study, a total of 45 individuals were selected for bronchoscopy (Figure 1). Participants were divided into two study groups: participants with COPD (never-smokers and ex-smokers) and never-smokers with normal lung function. Participants with COPD had postbronchodilator (post-BD) forced expiratory volume in 1 s (FEV_1_)/forced vital capacity (FVC) < 0.70 and FEV_1_/FVC z-score < −1.64 (<lower limit of normal, LLN) and FEV_1_ 50–100% of predicted value. Participants with normal lung function had post-BD FEV_1_/FVC ≥ 0.75, FEV_1_/FVC z-score of ≥ LLN and FEV_1_ ≥ 90% of predicted value. Reference values used were from the European Community for Coal and Steel cohort (ECCS) (12), and for LLN (z-score), according to the Global Lung Initiative (GLI) (13). Participants who had smoked fewer than 100 cigarettes or 20 cigars in their lifetime and had not smoked at all in the past 2 years were defined as “never-smokers.” Participants with a prior history of tobacco smoking, who had at least 10 pack-years of tobacco smoking and had been more than 2 years since they stopped smoking, were referred to as “ex-smokers” in this study.

Participants were excluded if they had an exacerbation requiring antibiotics or oral corticosteroids within 3 months or were using immunomodulating treatments. The participants were not allowed to use inhaled corticosteroids (ICS) for 3 months prior to bronchoscopy, and 1 month was required for common colds or viral upper airway infections. Other exclusion criteria included a history of significant cardiovascular diseases, coagulopathies, interstitial lung diseases, bronchiectasis, allergic eye/nose symptoms, and alcohol or drug abuse. A history of known causes of airway obstruction, such as alpha-1 antitrypsin deficiency or primary ciliary dyskinesia, was not allowed. In the study group with normal lung function, asthma was not allowed. Details of the exclusion criteria can be found in the Supplementary material.

### 2.2 Procedures before bronchoscopy

The study participants underwent three visits as part of the study protocol. The first visit was a screening visit, as previously described (11) for medical history, including respiratory symptoms, vital signs, routine blood tests, biobank samples and lung function tests. The participants received an economic compensation of 500 SEK (approximately 45 EUR or 50 USD) for the screening, along with 2,300 SEK (210 EUR and 230 USD) for the research bronchoscopy.

A preprocedural clinical visit with a respiratory physician was arranged before the bronchoscopy as a second visit. Then, a detailed medical history was repeatedly taken, and a physical examination was performed. Current medications were documented in the medical record. Information about premedication, monitoring, topical anesthesia, airway sampling, and potential risks was given. An electrocardiogram (ECG) was performed, as well as a chest X-ray if not done during the last 3 months. Premedication for bronchoscopy was prescribed and blood samples were collected.

### 2.3 Bronchoscopy and sampling of the airways

On the day of the bronchoscopy, participants were fasting. Before the bronchoscopy, research samples were collected: blood (a total of 50 mL), urine and a mouthwash for analysis of mediators.

During bronchoscopy, participants were in the supine position and monitored for ECG, heart rate, and oxygen saturation (SaO_2_) by transcutaneous pulse oximetry. Supplemental oxygen (1–2 L/min) was administered via nasal prongs if deemed necessary by the bronchoscopist. Topical anesthesia was provided following local guidelines. Intravenous midazolam [2–3 (−5) mg] and robinul [0.2 (−0.4) mg] were administered before and during the procedure based on the physician’s clinical judgment. Additional doses of midazolam and/or alfentanil were administered during the procedure if required; alfentanil was given in doses of 0.25–0.5 mg. Bronchoscopy was performed by senior consultants, all with many years of experience, using a bronchoscope with an external diameter of 5 mm via an oral approach. During the bronchoscopy, the following airway samples were collected: Bronchial wash with three 10 mL aliquots of phosphate-buffered saline (PBS) at pH 7.4, pre-warmed to 37°C, administered in the right upper lobe for microbial culture. Bronchial brush samples were optional for the site, with up to five samples collected from the airway tree. A total of 10 endobronchial mucosal biopsy samples were obtained from the bronchial tree. Bronchoalveolar lavage (BAL) was performed with 180 mL of PBS, administered in the middle lobe in three 60 mL portions. After bronchoscopy, the participants fasted for 2.5 h and were then offered coffee or tea and a sandwich before leaving the hospital. At one study center, oral corticosteroids following bronchoscopy were given (*n* = 5) according to local guidelines. Each bronchoscopy procedure lasted approximately 30 min.

### 2.4 Questionnaires

Before and after the bronchoscopy procedure, a questionnaire of five questions regarding health status was completed. The questions were answered on a 6-item Likert scale (0–5), with higher scores indicating a worse health status. The questions addressed symptoms of cough, phlegm (mucus), wheeze, chest tightness and energy levels. The questionnaire was administered by a research nurse at the clinic on the morning of the bronchoscopy and was repeated by telephone the day after the bronchoscopy.

Another set of questions was developed to examine participants’ experience of undergoing a research bronchoscopy. These questions were tested in a pilot study on 15 healthy volunteers (14) before being used in the present study. All questions regarding participants’ experiences were answered by telephone the day after the bronchoscopy.

### 2.5 Statistical analysis

The Statistical Package for the Social Sciences (SPSS, IBM, New York, United States) was used for the analyses. The clinical characteristics were summarized using descriptive quantitative statistics and presented as numbers (*n*), per cent (%), median and interquartile range (IQR), or mean and standard deviation (SD). Subgroup analyses were conducted based on sex and lung function.

## 3 Results

### 3.1 Participants clinical characteristics

Of the 58 eligible participants who first accepted to participate and underwent a pre-bronchoscopy visit at the clinic, 13 were excluded due to later declining participation (*n* = 5), comorbidity (*n* = 3), medication not allowed (*n* = 2) or other reasons (COVID-19, allergy, private reasons, *n* = 3). Finally, a total of 45 participants performed bronchoscopy and were included in the data analysis. Of those, 25 had COPD (15 never-smokers and 10 ex-smokers) and 20 had normal lung function.

In all participants, age ranged from 54 to 72 years with a mean of 60.5 years (±4.6), and 44% were women. Most of the participants (89%) had no prior experience with bronchoscopy (Table 1). Their mean oxygen saturation was 97.6%, and their mean body mass index (BMI) was 26.4 kg/m^2^. Nearly all had a high education level (high school or college, university), most lived in detached or semi-detached houses, and most of them were still working (78%). A minority of participants reported respiratory symptoms (18%) during the last year, mostly wheezing. Some had a productive cough in the last year (24%), and only 4% reported chronic cough. In descending order, the most common comorbidities were hypertension, depression, sleep apnea, pet allergy, asthma, diabetes, and other heart diseases.

**Table 1:** Clinical characteristics of all participants who performed bronchoscopy, divided into those with normal lung function and those with COPD.

Characteristics	All participants (*n* = 45)	Participants with normal lung function (*n* = 20)	Participants with COPD (*n* = 25)
Age, years, mean (±SD) (min–max)	60.5 (±4.6) (53.7–71.5)	59.9 (±4.0) (54.3–66.7)	61.0 (±5.1) (53.7–71.5)
Sex, *n* (%)			
Female	20 (44.4)	11 (55.0)	9 (36.0)
Male	25 (55.6)	9 (45.0)	16 (64.0)
Ex-smokers, *n* (%)	10 (22.2)	0 (0)	10 (40.0)
Never-smokers, *n* (%)	25 (77.8)	20 (100.0)	15 (60.0)
BMI, kg/m^2^ mean (±SD) (min–max)	26.4 (±3.9) (20.8–36.3)	25.6 (±3.8) (20.8–36.2)	27.1 (±3.9) (20.8–36.3)
First time bronchoscopy, *n* (%)	40 (88.9)	20 (100.0)	20 (80.0)
SaO_2_ (%), mean (±SD) (min–max)	97.6 (±1.4) (94.0–100.0)	98.1 (±1.4) (95.0–100.0)	97.2 (±1.4) (94.0–99.0)
FEV_1_ (% pred) post BD, mean (±SD) (min–max)	93.7 (±20.3) (56–143)	111.1 (±13.8) (91–143)	79.9 (±12.3) (56–100)
FEV_1_/FVC post BD, mean (±SD) (min–max)	0.68 (±0.11) (0.43–0.88)	0.79 (±0.04) (0.70–0.88)	0.59 (±0.06) (0.43–0.67)
Dyspnea, MRC scale, median (IQR)	0 (0-1)^a^	0 (0–1)	0 (0-1)^a^

Data are presented as median (IQR), mean ± SD, min–max, or numbers (%).BD, bronchodilator; BMI, body mass index; FEV_1_, forced expiratory volume in first second; FVC, forced vital capacity; IQR, interquartile range; MRC, Medical Research Council Scale; SaO_2_, oxygen saturation; SD, standard deviation.

a One missing value.

### 3.2 Experiences of bronchoscopy

Half of the participants found the overall experience of a research bronchoscopy a little unpleasant but okay (48%), simple (23%), or very simple (11%), and only 18% found it unpleasant. More women than men found it unpleasant (21% vs. 16%) and participants with normal lung function found it more unpleasant than participants with COPD (25% vs. 12%). Some experienced the bronchoscopy to be worse than expected (21%), but most of them found it to be better than expected (43%) or as expected (38%). The airway samplings were generally regarded as only a little unpleasant to very simple (89–91%). The samplings were found to be unpleasant or very unpleasant by more women than men (15% vs. 8%) and by more participants with normal lung function than participants with COPD (15% vs. 8%). Almost all participants experienced that the pre-medication had the intended effect (89%). Twenty per cent of all participants experienced receiving topical anesthesia as being unpleasant or very unpleasant, more among participants with normal lung function than among participants with COPD (25% vs. 16%). Participants were, to a large extent, satisfied with the information they received before the bronchoscopy.

By telephone interview, self-reported symptoms after bronchoscopy were chills (44%), fever (38%) and mild hemoptysis (32%). Half of the participants (53%) used paracetamol or ibuprofen after the procedure.

### 3.3 Reasons for participating in research bronchoscopy

Most participated in the research bronchoscopy to contribute to research (42%) or in combination with receiving a thorough examination of the lungs (38%). There were more women motivated by contributing to research than men (45% vs. 40%). Almost half of the participants (44%) were absolutely sure they would participate in a research bronchoscopy again if asked, another 44% said they might do it again, and only 11% would not participate again. Participants with normal lung function (15%) and women (20%) were the least interested in undergoing a research bronchoscopy again (Table 2).

**Table 2:** Participants’ experiences of participating in research bronchoscopy divided into women versus men, and participants with normal lung function versus COPD.

Questions on participants’ experiences of participating in a research bronchoscopy, *n* (%)	All participants (*n* = 45)	Women (*n* = 20)	Men (*n* = 25)	Participants with normal lung function (*n* = 20)	Participants with COPD (*n* = 25)
How satisfied are you with the information you received before the bronchoscopy? (scores 1–5)					
Very satisfied	42 (93.3)	19 (95.0)	23 (92.0)	19 (95.0)	23 (92.0)
Somewhat satisfied	3 (6.7)	1 (5.0)	2 (8.0)	1 (5.0)	2 (8.0)
Neutral	0 (0)	0 (0)	0 (0)	0 (0)	0 (0)
Unsatisfied	0 (0)	0 (0)	0 (0)	0 (0)	0 (0)
Very unsatisfied	0 (0)	0 (0)	0 (0)	0 (0)	0 (0)
Did the pre-medication have the intended effect?					
Yes	37 (88.1)^c^	17 (94.4)^b^	20 (83.3)^a^	16 (89)^b^	21 (88)^a^
How did you experience receiving anesthetic before the bronchoscopy? (scores 1–6)					
Very simple	5 (11.1)	1 (5.0)	4 (16.0)	2 (10.0)	3 (12.0)
Simple	7 (15.6)	1 (5.0)	6 (24.0)	5 (25.0)	2 (8.0)
A little unpleasant, but okay	24 (53.3)	14 (70.0)	10 (40.0)	8 (40.0)	16 (64.0)
Unpleasant	6 (13.3)	2 (10.0)	4 (16.0)	3 (15.0)	3 (12.0)
Very unpleasant	3 (6.7)	2 (10.0)	1 (4.0)	2 (10.0)	1 (4.0)
The worst experience ever	0 (0)	0 (0)	0 (0)	0 (0)	0 (0)
How did you experience the bronchoalveolar lavage (BAL)? (scores 1–6)					
Very simple	19 (42.2)	5 (25.0)	14 (56.0)	6 (30.0)	13 (52.0)
Simple	10 (22.2)	7 (35.0)	3 (12.0)	7 (35.0)	3 (12.0)
A little unpleasant, but okay	11 (24.4)	5 (25.0)	6 (24.0)	4 (20.0)	7 (28.0)
Unpleasant	4 (8.9)	2 (10.0)	2 (8.0)	3 (15.0)	1 (4.0)
Very unpleasant	1 (2.2)	1 (5.0)	0 (0)	0 (0)	1 (4.0)
The worst experience ever	0 (0)	0 (0)	0 (0)	0 (0)	0 (0)
How did you experience the biopsy sampling? (scores 1–6)					
Very simple	20 (44.4)	8 (40.0)	12 (48.0)	6 (30.0)	14 (56.0)
Simple	12 (26.7)	4 (20.0)	8 (32.0)	5 (25.0)	7 (28.0)
A little unpleasant, but okay	9 (20.0)	6 (30.0)	3 (12.0)	7 (35.0)	2 (8.0)
Unpleasant	2 (4.4)	2 (10.0)	2 (8.0)	2 (10.0)	2 (8.0)
Very unpleasant	2 (4.4)	0 (0)	0 (0)	0 (0)	0 (0)
The worst experience ever	0 (0)	0 (0)	0 (0)	0 (0)	0 (0)
Overall, how did you experience the bronchoscopy examination? (scores 1–6)					
Very simple	5 (11.4)	1 (5.3)	4 (16.0)	2 (10.0)	3 (12.0)
Simple	10 (22.7)	2 (10.5)	8 (32.0)	4 (20.0)	6 (24.0)
A little unpleasant, but okay	21 (47.7)	12 (63.2)	9 (36.0)	9 (45.0)	12 (48.0)
Unpleasant	8 (18.2)	4 (21.1)	4 (16.0)	5 (25.0)	3 (12.0)
Very unpleasant	0 (0)	0 (0)	0 (0)	0 (0)	0 (0)
The worst experience ever	0 (0)	0 (0)	0 (0)	0 (0)	0 (0)
Did you get a fever after the bronchoscopy?					
yes	17 (37.8)	6 (30.0)	11 (44)	6 (30)	11 (44)
Did you get chills after the bronchoscopy?					
yes	20 (44.4)	9 (45)	11 (44)	8 (40)	12 (48)
Did you take any medicine after the bronchoscopy?					
yes	24 (53.3)^a^	12 (63.2)^a^	12 (48.0)	8 (40.0)	16 (66.7)^a^
Have you coughed up blood after the bronchoscopy?					
yes	14 (31.8)^a^	6 (31.7)^a^	8 (32.0)	7 (35)	7 (29.2)^a^
How did you experience the bronchoscopy in relation to what you had expected? (scores 1–5)					
Much better	7 (15.6)	2 (10.0)	5 (20.0)	3 (15.0)	4 (16.0)
Better	12 (26.7)	5 (25.0)	7 (2.0)	3 (15.0)	9 (36.0)
As expected	17 (37.8)	8 (40.0)	9 (36.0)	9 (45.0)	8 (32.0)
Somewhat worse	6 (13.3)	4 (20.0)	2 (8.0)	4 (20.0)	2 (8.0)
Much worse	3 (6.7)	1 (5.3)	2 (8.0)	1 (5.0)	2 (8.0)
Why did you say yes to participating in the study?					
Contribute to research	19 (42.2)	9 (45.0)	10 (40.0)	8 (40.0)	11 (44.0)
Get a thorough lung examination	7 (15.6)	6 (30.0)	1 (4.0)	3 (15.0)	4 (16.0)
Contribute to research + to get a thorough lung examination	17 (37.8)	5 (25.0)	12 (48.0)	9 (45.0)	8 (32.0)
Financial compensation + to get a thorough lung examination	2 (4.4)	0 (0)	2 (8.0)	0 (0)	2 (8.0)
Financial compensation	0 (0)	0 (0)	0 (0)	0 (0)	0 (0)
Do not know	0 (0)	0 (0)	0 (0)	0 (0)	0 (0)
Would you consider participating in a bronchoscopy study again? (scores 1–3)					
Yes, absolutely	20 (44.4)	7 (35.0)	11 (44.0)	9 (45.0)	11 (44.0)
Maybe	20 (44.4)	9 (45.0)	13 (52.0)	8 (40.0)	12 (48.0)
No, absolutely not	5 (11.1)	4 (20.0)	1 (4.0)	3 (15.0)	2 (8.0)

Data are shown as *n*; *n*, numbers; %, per cent. COPD, chronic obstructive pulmonary disease.

a One missing value.

b Two missing values.

c Three missing values.

### 3.4 Impact of research bronchoscopy

The participant’s health status did not change after bronchoscopy regarding wheezing, chest tightness and energy levels (Table 3). However, increased cough and phlegm were reported. There were no differences found regarding cough, tightness/pressure of the chest, or wheeze in the chest between participants with normal lung function and participants with COPD before and after bronchoscopy. Participants with normal lung function reported more phlegm after bronchoscopy compared to participants with COPD.

**Table 3:** Health status before and after a research bronchoscopy, shown for all participants and divided into groups of participants with normal lung function or COPD.

Questions regarding health status presented with responses given before and after bronchoscopy, scores range from 0 to 5, median (range)	Participants		
	All (*n* = 45)	With normal lung function (*n* = 20)	With COPD (*n* = 25)
Cough			
“I never cough—I cough all the time”			
Before	0 (0–3)	0 (0–2)	0 (0–3)
After	1 (0–3)	1 (0–3)	1 (0–3)
Phlegm (mucus) in chest			
“I have no phlegm in my airways—my airways are full of phlegm”			
Before	0 (0–3)	0 (0–2)	1 (0–3)
After	1 (0–4)	1 (0–3)	1 (0–4)
Tightness/pressure of chest			
“My chest does not feel tight at all—my chest feels very tight”			
Before	0 (0–3)	0 (0–3)	0 (0–1)
After	0 (0–4)	0 (0–3)	0 (0–4)
Wheeze in the chest			
“I do not have wheeze in the chest—I have wheeze in the chest even at rest”			
Before	0 (0–1)	0 (0–1)	0 (0–1)
After	0 (0–2)	0 (0–1)	0 (0–2)
Level of energy			
“I have lots of energy—I have no energy at all”			
Before	1 (0-4)^a^	1 (0-3)^a^	1 (0-4)^a^
After	1 (0-5)^a^	0 (0-4)^a^	1 (0–5)

Questions with answer scores ranging from 0 to 5 presented with median and range. COPD, chronic obstructive pulmonary disease; *n*, numbers.

a One missing value.

## 4 Discussion

The overall experience of undergoing a bronchoscopy in a research setting was generally considered tolerable, with only a few participants finding it unpleasant and most reported their experience to be much better or as expected. Participants were largely satisfied with the information they received before the bronchoscopy. The sampling procedures were mostly perceived as either slightly unpleasant or very simple. Notably, more women than men reported the samplings as unpleasant or very unpleasant, as did more participants with normal lung function compared to those with COPD.

Bronchoscopy is a safe procedure with a low complication rate (15). In this study, the participants reported chills, fever, and mild hemoptysis. The post-bronchoscopy complications were in accordance with previous reports (1), but somewhat difficult to fully compare since definitions of complications and discomfort vary between studies. Complications previously reported in patients undergoing bronchoscopy include a number of symptoms and signs such as hypoxia, pneumothorax, tachycardia, hemorrhage (9), coughing, hemodynamic changes and panic/subject unease (2, 9). Almost all participants experienced the intended effects of the pre-medication, but because of that, some participants could have been too “tired” to recall the procedure when asked about their experiences. This has been addressed in a qualitative study on participants with severe COPD, where half of them were unaware due to sedation during the bronchoscopy, and their reflections were mostly positive. They talked about drifting away and described being “knocked out” by the drugs and not remembering what happened (16). The tiredness could be challenging when answering the questions regarding their experiences, as they might not be fully aware of the various aspects of the procedure while sedated. It has been shown in a study on participants with COPD that those undergoing bronchoscopy with conscious sedation were often fully aware of the procedure. Many remembered feeling discomfort, especially from coughing and choking, which caused them distress (16). Discomfort during the bronchoscopy procedure has been shown to be an important factor affecting patient satisfaction (3).

In this study, the participants received pre-medication with midazolam and intravenous glycopyrronium and additional doses of midazolam and/or alfentanil when needed, according to the bronchoscopist’s clinical judgment. A greater comfort and satisfaction for patients has been described when the bronchoscopy was performed intubated under monitored anesthesia compared to when the bronchoscopy was performed under only topical anesthesia (4). This was also recognized in the present study as 88% experienced effect of the pre-medication, even though some (20%) had an unpleasant or very unpleasant experience of receiving the topical anesthesia. In a recent pilot study on healthy volunteers, research bronchoscopies without sedation were investigated. It was shown that minimizing the need for sedation by only using topical anesthesia has the potential to improve patient safety and enhance the tolerability of the procedure (17), which might also improve participants’ recollection of the procedure. Participants were mostly very satisfied with the information given before the bronchoscopy, and only 20% found the experience to be worse than expected. The importance of participants’ knowledge regarding the procedure has been shown to be significant. Providing adequate and relevant information about the bronchoscopy procedure, including illustrations and pictures, has been shown to help to reduce potential anxiety and improve the tolerability of participants (18, 19). Reducing the duration of the procedure is another crucial aspect that influences the patient’s comfort (19). This study limited the procedure to approximately 30 min. Study participants have previously expressed concerns about the bronchoscopy procedure being time-consuming and interrupting their work schedule (5).

Most participants expressed a desire to contribute to research, a motive that was particularly prevalent among women. Previous research on bronchoscopy has shown that altruistic motives are more common in women than in men (8). In the present study, more women were unwilling to participate in further research bronchoscopy than men. This reluctance may stem from the fact that women experienced the bronchoscopy procedure more negatively than they had anticipated. They also found receiving the topical anesthesia more difficult compared to the men. A moderate level of anxiety before the procedure has also been shown to be more common among women than men (3). Fear of discomfort and concerns about study participation have been noted as common reasons for individuals who are eligible but choose not to participate in a research bronchoscopy (8).

There were some differences between the subgroups when it came to their experiences of the procedure. Only a small number found the administration of topical anesthesia unpleasant or very unpleasant, with a higher percentage of participants with normal lung function reporting discomfort compared to those with COPD. The airway sampling was also perceived as more unpleasant among those with normal lung function compared to those with COPD. The reason participants with COPD experienced less discomfort is unknown, but it is possible that they are more used to having respiratory symptoms. The willingness to return for a research bronchoscopy was high, with participants with COPD being the most motivated. Willingness to return for a research bronchoscopy was also addressed in another study, where the participants with COPD were slightly less willing to return for another research bronchoscopy than those in the control group (76% vs. 84%) (2), which was opposite to the present results. A review has, however, shown no difference between these groups regarding the willingness to repeat bronchoscopy (20).

A strength of this study was that the bronchoscopy procedure was performed by experienced bronchoscopists and healthcare teams, which limits variability in procedure performance and enables comparison despite several study sites and several bronchoscopists. The experience of the physician performing the bronchoscopy has also been shown to be an important factor in reducing the number of complications (21). In the present study, there were no serious complications reported, which was seen as a strength, but this could be because the participants with COPD included in this study had milder airflow limitation than in other studies (2, 8). Also, more never-smoking participants with COPD, who often have milder symptoms (22), were included in this study. It might influence participants’ fear and concerns positively before undergoing a research bronchoscopy to know that the bronchoscopist is experienced, but previous studies have found no correlation between patient satisfaction and the bronchoscopist’s experience (23).

A limitation worth recognizing was that the questionnaires used to assess participants’ experiences have not been validated in a research setting, although pilot tested. Questions used to assess health status were modified from the COPD Assessment Test (CAT), which is validated in COPD (24). The absence of formal validation may impact the reliability and interpretation of the findings. Future research would benefit from employing fully validated questionnaires. Adopting a qualitative study approach could provide a deeper understanding of participants’ experiences (25). Another limitation might be that participants answered questions by telephone the day after the bronchoscopy procedure, which may also reduce their ability to remember details of the procedure. However, a telephone interview is common practice when it comes to distributing questionnaires about participants’ experiences. When recruiting participants for the present study, the current restrictions due to the COVID-19 pandemic limited the number of participants available or willing to participate. To obtain a representative sample, the inclusion period was prolonged, but during this time, some participants declined to participate despite showing an initial interest. Due to the lack of follow-ups on participants’ reasons for declining, this information remains unknown, which is considered a limitation. The limited number of participants is also a limitation, as a larger group would have enabled more general conclusions.

## 5 Conclusion

The present study shows participants’ experiences of a bronchoscopy in a research setting, including well-informed participants, experienced bronchoscopists and experienced healthcare teams, to be tolerable for participants both with normal lung function and with COPD.

## Data Availability

The original contributions presented in the study are included in the article/Supplementary material, further inquiries can be directed to the corresponding author.

## Footnotes

^1^ https://www.clinicaltrials.gov/study/NCT03049202
