# Supplementary material for: Swedish study participants undergoing research bronchoscopy – a tolerable or unpleasant experience?
Source: Front Med (Lausanne). 2025 Sep 12;12:1648729. doi: 10.3389/fmed.2025.1648729 (PMC12465911; doi:10.3389/fmed.2025.1648729)
Supplement: Supplementary file 1 [file Table_1.DOCX]

**General Exclusion Criteria:**

1. Treatment with immunomodulating agents: Including systemic steroids and PDE4 inhibitors (nasal and ocular steroids allowed).
2. Treatment with inhaled corticosteroids: Within the last 3 months before bronchoscopy.
3. Conditions that may interfere with the studied diseases: As judged by the investigator (should be registered).
4. Exacerbation requiring oral corticosteroids and/or antibiotics: Within the last 3 months before bronchoscopy.
5. Two or more exacerbations within the last year: Requiring antibiotics and/or oral corticosteroids. Check question 37 in Broncho-SCAPIS questionnaire.
6. Common cold or viral upper airway infection: During the last month before bronchoscopy.
7. Known alcohol and/or drug abuse.
8. Significant ischemic or other cardiovascular disease.
9. History of significant coagulopathies and thromboembolic disease.
10. Treatment with warfarin or similar medications: (e.g., NOAK).
11. Salicylic acid as secondary prophylaxis: Salicylic acid as primary prophylaxis is allowed but should be terminated 5 days before bronchoscopy.
12. Significant other disease: That increases the risk for performing bronchoscopy, as judged by the investigator.
13. Alpha-1 antitrypsin deficiency: With genetic analysis showing PiZZ (PiMZ and PiSZ can be included).

**Group-Specific Exclusion Criteria:**

- **Exclusion Criteria for participants with COPD:**

Known causes of airway obstruction, as assessed by the investigator, including but not limited to alpha-1-antitrypsin deficiency, primary ciliary dyskinesia, significant bronchiectasis, interstitial lung diseases, and post-TB status.

- **Exclusion Criteria for participants with normal lung function**:

Asthma: The answer to question 25 (“Have you ever had asthma?”) in the Broncho-SCAPIS questionnaire must be “No”
